# Colonic metastasis of hepatocellular carcinoma with repeated retroperitoneal bleeding: a case report

**DOI:** 10.1186/s40792-021-01349-7

**Published:** 2021-12-18

**Authors:** Wataru Miyauchi, Manabu Yamamoto, Makinoya Masahiro, Yuji Shishido, Kozo Miyatani, Tomoyuki Matsunaga, Teruhisa Sakamoto, Yoshiyuki Fujiwara

**Affiliations:** Division of Gastrointestinal and Pediatric Surgery, Department of Surgery, School of Medicine, Faculty of Medicine, Tottori University, Yonago, 683-8504 Japan

**Keywords:** Hepatocellular carcinoma, Colon metastasis, Retroperitoneal hematoma

## Abstract

**Background:**

Colonic metastasis is uncommon in patients with hepatocellular carcinoma (HCC). In the past, extrahepatic metastasis of HCC was not treated aggressively because of its poor prognosis. Herein, we describe the case of a patient with HCC who survived for 30 months following resection of a metastatic tumor in the ascending colon.

**Case presentation:**

An 80-year-old man presented at our hospital with symptoms of abdominal pain on the right side and fever. He had undergone transcatheter arterial chemoembolization and posterior segment resection of the liver because of HCC, followed by radiofrequency ablation for a recurrent intrahepatic lesion 5 and 3 years, respectively, prior to the visit. He was diagnosed with retroperitoneal hematoma, which was thought to be associated with diverticulitis and an extramural tumor in the ascending colon. A definitive diagnosis could not be reached; however, a right hemicolectomy of the colon was performed because of progression to anemia. A pathological examination revealed a metastatic tumor in the ascending colon extending from the subserosal layer to the muscularis propria layer. The patient was treated with lenvatinib after surgery, but presented with intrahepatic recurrence, lymph node metastasis, and peritoneal dissemination metastasis 15 months later. The progression of the disease could not be controlled and his postoperative survival time was 30 months.

**Conclusion:**

Resection of metastasis of HCC might contribute to prolonged survival in cases, where radical resection is possible.

## Background

Colonic metastasis is an uncommon occurrence in hepatocellular carcinoma (HCC). In the past, extrahepatic metastasis of HCC was not treated aggressively because of its poor prognosis; however, recent studies indicate that surgery can improve the prognosis if the metastatic lesion is completely resected [1–3]. In this report, we describe the case of patient with HCC who survived for 30 months following the resection of a metastatic lesion in the ascending colon. In addition, a short description of the literature review is provided.

## Case presentation

An 80-year-old man visited a primary care physician, complaining of pain in the lower abdomen on the right side and fever An evaluation of the blood sample showed an increased inflammatory response, and abdominal ultrasonography revealed fluid retention in the right side of the abdomen. After referral to our hospital, a computed tomography (CT) scan was performed and the fluid accumulation in the abdomen was diagnosed as a retroperitoneal hematoma.

Previously, the patient had been followed up at our hospital for chronic hepatitis C. In addition, he had undergone transcatheter arterial chemoembolization (TACE) and posterior segment resection of the liver for HCC (S6; Couinaud’s hepatic segment) 5 years before this visit and radiofrequency ablation (RFA) for intrahepatic recurrence (S8) 3 years ago. The pathological findings of primary HCC are as follows. It was nodular type and showed expansive growth, fibrous capsule formation, capsule invasion, and septum formation. There was no serous membrane invasion, vascular invasion, and bile duct invasion. The liver in the non-tumor area had chronic hepatitis, and surgical margin was negative.

He also achieved a sustained virological response to hepatitis C virus with Ledipasvir acetonate / Sofosbuvir 2 years ago. The patient’s medical history included surgery for appendicitis, the use of stents for acute myocardial infarction, transurethral resection of a tumor in the bladder, hypertension, hypercholesterolemia, hyperuricemia, and osteoporosis. His medications included aspirin, losartan potassium, fenofibrate, benzbromarone, alendronate sodium hydrate, a proton pump inhibitor, and laxatives.

**Fig. 1:**
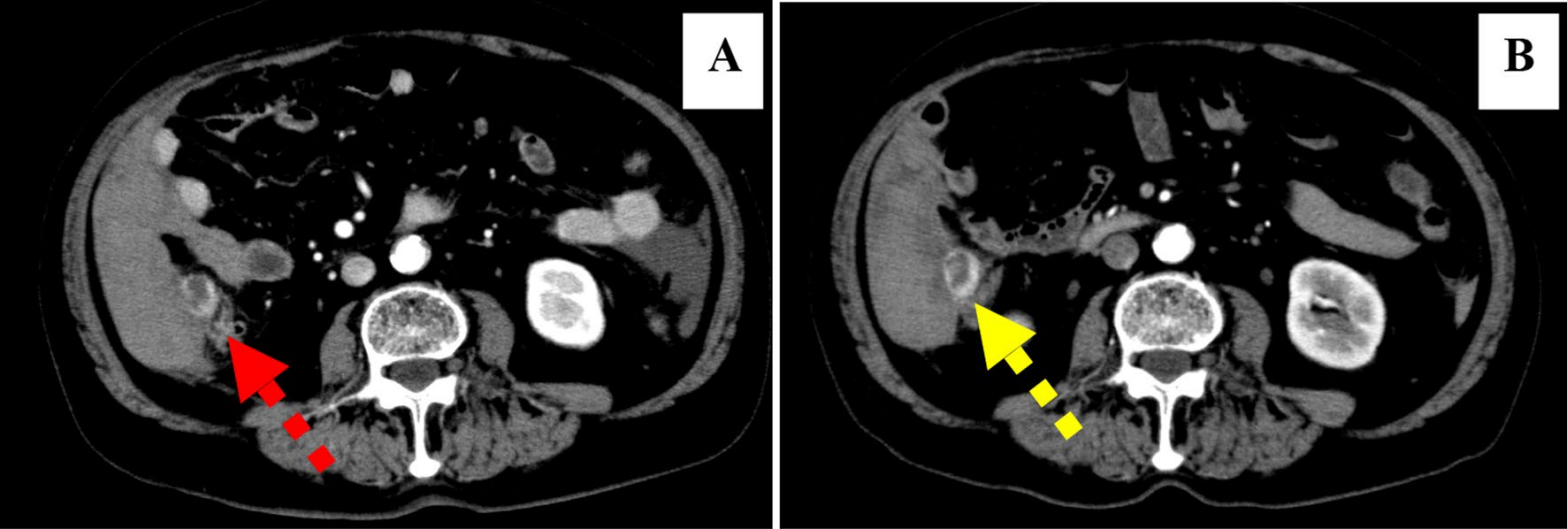
Preoperative contrast-enhanced CT. **A** CT image of the patient when he first came to the hospital with abdominal pain. **B** Preoperative CT image when the abdominal pain recurred 1 week after (**A**). A ring-shaped contrast area (arrows) between the ascending colon and the retroperitoneal hematoma. The anemia had worsened, but the size of the hematoma had not changed significantly

The patient’s blood pressure and pulse rate were stable, and mild anemia (hemoglobin [Hb] level, 9.8 g/dL) was observed during the first visit at our hospital; therefore, we assumed that the bleeding had stopped, and the patient was admitted to the hospital for follow-up. The symptoms seemed to improve for a while, but 1 week later, the patient experienced abdominal pain. The preoperative blood test showed a decrease in the Hb level to 8.2 g/dL. Contrast-enhanced CT at the time of visit showed a retroperitoneal hematoma centered in the right paracolic gutter (Fig. 1A). A ring-shaped contrast area was observed between the hematoma and the ascending colon. As colonic diverticula were also present, bleeding from the extramural tumor or diverticula was considered. Although there was no obvious enlargement of the hematoma (Fig. 1B), abdominal pain recurred in a short time and anemia progressed. Based on the above, it was judged that the bleeding could not be controlled by follow-up, and surgery was decided upon. Preoperative liver function was as follows. Total protein 6.4 g/dL, Albumin 3.5 g/dL, Total bilirubin 1.4 mg/dL, Direct bilirubin 0.4 mg/dL, Aspartate aminotransferase 33 U/L, Alanine aminotransferase 23 U/L, γ-glutamyl transpeptidase 104 U/L, Cholinesterase 166 U/L, Lactate dehydrogenase 240 U/L. Tumor markers were not measured preoperatively.

### Surgical findings

**Fig. 2:**
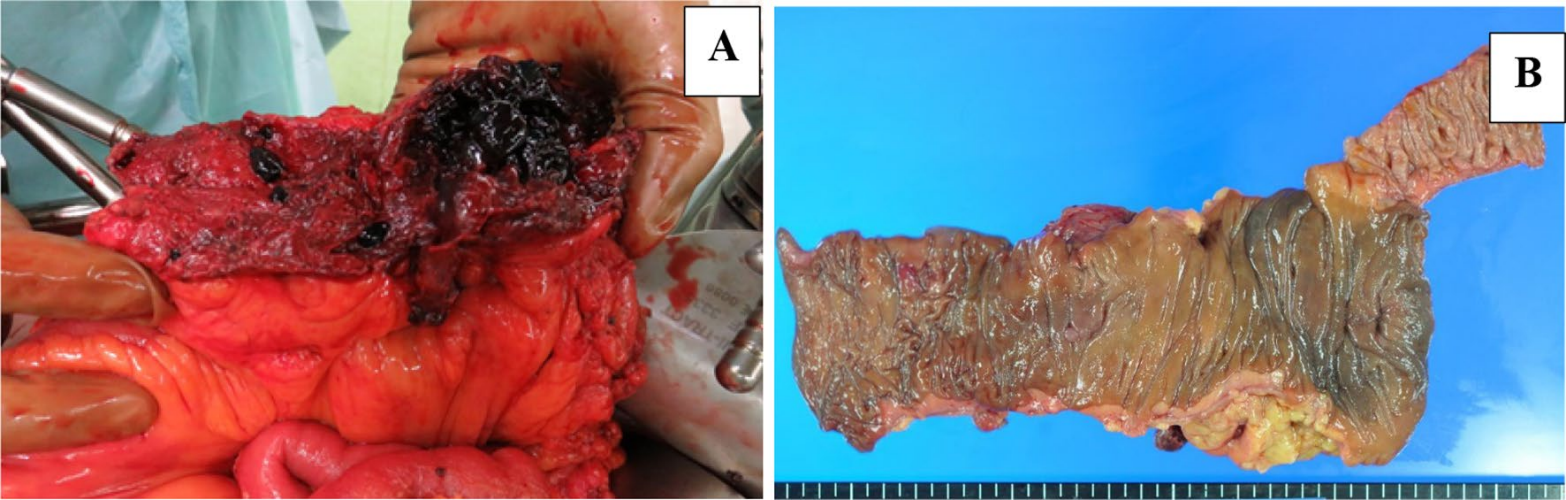
Surgical findings. **A** Hematoma extending from the ascending colon to the retroperitoneum. **B** There were no abnormal findings on the mucosa

On laparotomy, a small amount of bloody ascites was observed from the pelvis to the left lower abdomen, but no peritoneal dissemination lesion was detected. Strong adhesions were found on the right side of the abdomen; the removal of these adhesions resulted in a retroperitoneal hematoma. The cause of the bleeding and he location of the tumor could not be identified intraoperatively because of the extensive hematoma. A hemicolectomy was performed on the right side, because we judged from the preoperative CT that the hematoma and tumor could be resected together (Fig. 2). The total time of the operation was 217 min and the blood loss was estimated at 505 mL.

### Pathological findings

**Fig. 3:**
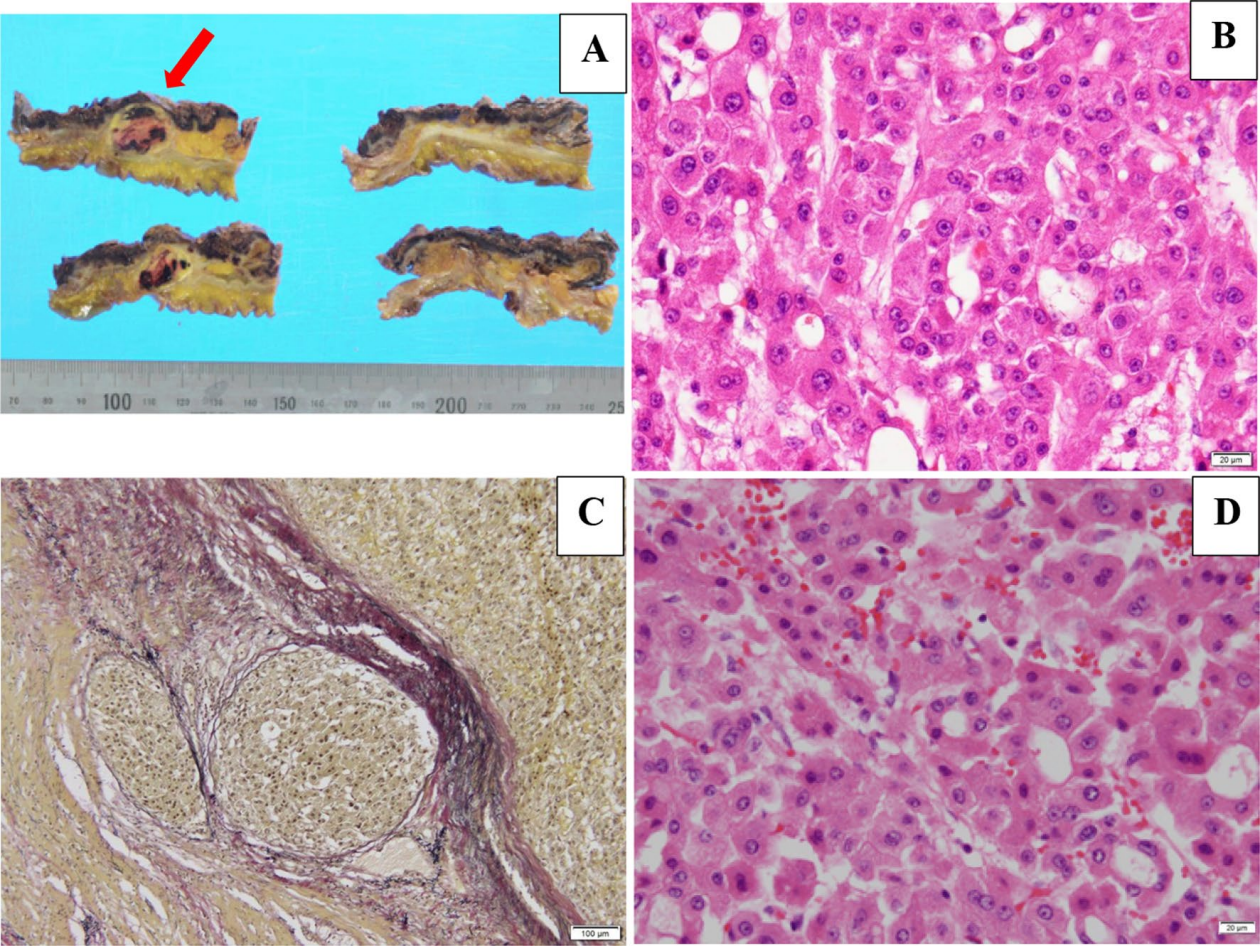
Macroscopic and microscopic findings of the metastatic tumor in the ascending colon. **A** Macroscopic findings of the metastatic tumor. Colonic metastasis (Φ20 mm, red arrow) was mainly observed within the subserosal and muscularis layers in the ascending colon, along with a contiguous retroperitoneal hematoma. **B** Microscopic findings revealed a moderately differentiated type of HCC. **C** The Elastica-van Gieson stain showed vascular invasion of the metastatic tumor. **D** Microscopic findings of primary HCC

The patient was diagnosed with moderately differentiated HCC of the ascending colon, located mainly within the subserosal and muscular layers, where a continuous retroperitoneal hematoma was observed. This tumor was similar to the tumor resected 5 years ago and was diagnosed as metastasis of the ascending colon (Fig. 3). The Elastica-van Gieson stain showed vascular invasion of the metastatic tumor, which was considered to be hematogenous metastasis.

### Postoperative course

The patient resumed drinking water and taking oral medications on the first postoperative day (POD). Paralytic bowel obstruction occurred during the postoperative course, but improved with conservative treatment, and the patient was discharged home on the 20th POD without any other complications.

After the patient was discharged from the hospital, he was followed up without postoperative adjuvant chemotherapy at his own request. However, 6 months after surgery, CT showed peritoneal dissemination, and lenvatinib 8 mg was started on a daily administration as a patient with unresectable HCC. Because of side effects such as decreased appetite, the dose was reduced and the administration interval was spaced accordingly. However, 15 months after surgery, he presented with intrahepatic, lymph node recurrence. The same chemotherapy was continued thereafter, but the progression of the disease could not be controlled. 26 months after surgery, he was switched to palliative care and his postoperative survival time was 30 months.

## Discussion

Colonic metastasis of HCC is an uncommon occurrence. According to the Report of the 21st Nationwide Follow-Up Survey of Primary Liver Cancer in Japan (2010–2011) [4], extrahepatic recurrence was observed in 1189 (5.86%) of 20,284 registered cases of HCC. Among them, the lungs, lymph nodes, and bone were the most commonly involved. Gastrointestinal metastasis has been reported in 0.5–4% of patients [5], but most of them were direct invasions of the stomach and duodenum [6]. Metastasis to the colon is rare, because compared to the upper gastrointestinal tract, such as the duodenum, the colon is not in contact with the liver; thus, the possibility of a direct invasion is low.

Initially, the implantation of tumor cells by RFA was suspected in our patient. Subsequently, it was considered hematogenous metastasis, because the site of the lesion was away from the puncture site, the main locus of the metastatic lesion was limited to the subserosal and muscular layers, and vascular invasion was observed.

In the case of hematogenous metastasis, the venous flow from the colon to the liver is reversed, possibly because of the increase in the portal pressure in patients with cirrhosis and during TACE. Table 1 shows a summary of patients with colon metastasis from HCC obtained after a literature search in PubMed using the keywords “hepatocellular carcinoma” and “colon metastasis.” Several cases of hematogenous metastasis after TACE were reported [10, 15, 16]. In this case, there was no portal vein tumor thrombosis in his treatment history, but the patient had undergone TACE, which may have led to hematogenous metastasis. The median time from initial HCC to colorectal metastasis is 30 months (Table 1), so continuous follow-up is necessary. Although the median survival time after colorectal metastasis in the cases reported in the literature is 5.5 months, some studies have reported longer periods of over 1 year (Table 1). In most of these cases, surgery was performed to control bleeding rather than to cure the cancer. However, aggressive resection of the metastasis may lead to a prolonged prognosis if the general condition is acceptable and complete resection is possible.

**Table 1:** Summary of patients with colon metastasis from HCC (data obtained from the literature search)

Case	year	Author	Age	Sex	Underlying liver disease	Previous treatment	Time from initial HCC to colorectal metastasis	Site of metastasis	Treatment for colon metastasis	Survival period after colon metastasis
1	1999	Cosenza et al. [7]	82	Female	Hepatitis C	Surgery, chemotherapy	4 years	Ascending colon	Surgery	Unknown
2	2007	Tapuria et al. [8]	67	Male	Autoimmune chronic active hepatitis	Immunosuppression	Unknown	Ascending colon	Bypass operation	Over 1 month
3	2008	Hirashita et al. [9]	79	Male	Hepatitis C	TACE	1.5 years	Transverce colon	Surgery	6 months
4	2008	Nozaki et al. [10]	69	Male	Unknown	Surgery	2 years	Ascending colon	BSC	Less than 1 month
5	2010	Yoo et al. [11]	47	Male	Hepatitis B	TACE	1.5 years	Sigmoid colon	Surgery	Over 4 months
6	2011	Huang et al. [12]	57	Female	Hepatitis C	Surgery	1.5 years	Rectum	EMR	Unknown
7	2014	Miyaki et al. [13]	79	Male	Hepatitis B	Surgery, TACE, chemotherapy	12 years	Sigmoid colon, stomach, lip	EMR	Over 2 years
8	2014	Ou et al. [5]	62	Male	Hepatitis B	Surgery, RFA, PEI, Cyberknife stereotactic radiosurgery, TACE	3 years	Ascending colon, rectum	EMR	1 month
9	2016	Wu et al. [14]	54	Male	Unknown	Surgery	5 years	Ascending colon, stomach	Surgery	2 years
10	2016	Ikeda et al. [15]	82	Female	Liver cirrhosis	RFA, TACE	4 months	Rectum	Surgery	5 months
11	2016	Zhu et al. [16]	47	Male	Hepatitis B	Surgery, TAE, TACE	5 years	Transverse colon	Surgery	Over 1 year
12	2017	Tanaka et al. [6]	60	Female	Normal Liver	―	Simultaneous	Transverse colon	Surgery	Over 1 year
13	2017	Nakajima et al. [17]	75	Male	Normal Liver	Surgery	7 months	Transverse colon	Surgery	1 year
14	2019	Tagliabue et al. [3]	70	Male	Hepatitis B	TACE	Unknown	Sigmoid colon	Surgery	Unknown
15	2019	Pham et al. [18]	60	Male	Hepatitis B	TACE	Unknown	Sigmoid colon	Surgery	Unknown
16	2020	Yu et al. [1]	60	Male	Hepatitis B	Surgery, TACE, RFA, Chemotherapy	10 years	Sigmoid colon	Surgery	Unknown
17	2020	Kim et al. [19]	75	Male	alcoholic Liver cirrhosis	Surgery, TACE	3 years	Ascending colon, stomach, Brain	Surgery	1.5 months
18	2021	Our case	80	Male	Hepatitis C	Surgery, TACE, RFA	5 years	Ascending colon	Surgery	2.5 years

*BSC* best supportive care, *EMR* endoscopic mucosal resection, *PEI* percutaneous ethanol injection, *RFA* radiofrequency ablation, *TACE* transcatheter arterial chemoembolization, *TAE* transcatheter arterial embolization

According to the Japanese guidelines for the treatment of HCC [20], the administration of molecular targeted drugs is strongly recommended to treat extrahepatic metastases of HCC, whereas local therapy, such as resection, is only weakly recommended when intrahepatic lesions are absent or well controlled. Extrahepatic recurrence is often associated with multiple lesions [21]. In this case study, no recurrent sites were observed in the liver and no other metastases were detected in the abdomen. Despite the absence of a definitive diagnosis, in this case, surgery was performed to control the bleeding. In one study, the 1-year cumulative survival rate was reported to be 20% and the 3-year cumulative survival rate in the extrahepatic metastasis non-resection group was not reported [22]. However, our patient survived for 30 months, and resection of the metastases might have contributed to the improved prognosis. Of course, if there are no symptoms that require surgical treatment, such as bleeding, chemotherapy may be able to prolong survival. However, more cases need to be collected and examined to determine which is better.

When a ruptured tumor accompanied by retroperitoneal hemorrhage was found, HCC colonic metastasis should be ruled out. Although a preoperative diagnosis was not possible in this case, the use of Tc-99 m for the diagnosis of colorectal metastasis of HCC has been reported previously [23], which might prove useful for diagnosis when extrahepatic metastasis of HCC is suspected based on the patient’s medical history.

## Conclusions

Resection of metastases of HCC contributed to a prolonged prognosis in the patient in this study and might prove useful in cases, where radical resection is possible.

## Data Availability

All data regarding this paper are available on request.

## Abbreviations

CT: Computed tomography
Hb: Hemoglobin
HCC: Hepatocellular carcinoma
Hct: Hematocrit
POD: Postoperative day
RBC: Red blood cell
RFA: Radiofrequency ablation
TACE: Transcatheter arterial chemoembolization
